# Association of Oscillatory Ventilation during Cardiopulmonary Test to Clinical and Functional Variables of Chronic Heart Failure Patients

**DOI:** 10.21470/1678-9741-2017-0158

**Published:** 2018

**Authors:** Hugo Valverde Reis, Priscila Abreu Sperandio, Clynton Lourenço Correa, Solange Guizilini, José Alberto Neder, Audrey Borghi-Silva, Michel Silva Reis

**Affiliations:** 1 Research Group in Cardiorespiratory Rehabilitation (GECARE) and Department of Physical Therapy, Faculdade de Medicina, Universidade Federal do Rio de Janeiro (UFRJ), Rio de Janeiro, RJ, Brazil.; 2 Pulmonary Function and Clinical Exercise Physiology Unit (SEFICE), Respiratory Division, Department of Medicine, Escola Paulista de Medicina, Universidade Federal de São Paulo (EPM-UNIFESP), São Paulo, SP, Brazil.; 3 Respiratory Division, Department of Physiotherapy, Universidade Federal de São Paulo (UNIFESP), São Paulo, Brazil.; 4 Laboratory of Cardiopulmonary Physical Therapy (LACAP), Department of Physical Therapy, Universidade Federal de São Carlos (UFSCAR), São Carlos, SP, Brazil.

**Keywords:** Heart Failure, Exercise Test, Respiratory Mechanics, Physical Exertion

## Abstract

**Objective:**

The aim of this study is to characterize the presence of exercise oscillatory ventilation (EOV) and to relate it with other cardiopulmonary exercise test (CET) responses and clinical variables.

**Methods:**

Forty-six male patients (age: 53.1±13.6 years old; left ventricular ejection fraction [LVEF]: 30±8%) with heart failure were recruited to perform a maximal CET and to correlate the CET responses with clinical variables. The EOV was obtained according to Leite et al. criteria and VE/VCO_2_ > 34 and peak VO_2_ < 14 ml/kg/min were used to assess patients' severity.

**Results:**

The EOV was observed in 16 of 24 patients who performed the CET, as well as VE/VCO_2_ > 34 and peak VO_2_ < 14 ml/kg/min in 14 and 10 patients, respectively. There was no difference in clinical and CET variables of the patients who presented EOV in CET when compared to non-EOV patients. Also, there was no difference in CET and clinical variables when comparing patients who presented EOV and had a VE/VCO_2_ slope > 34 to patients who just had one of these responses either.

**Conclusion:**

The present study showed that there was an incidence of patients with EOV and lower peak VO_2_ and higher VE/VCO_2_ slope values, but they showed no difference on other prognostic variables. As well, there was no influence of the presence of EOV on other parameters of CET in this population, suggesting that this variable may be an independent marker of worst prognosis in HF patients.

**Table:** 

Abbreviations, acronyms & symbols				
ACE	= Angiotensin-converting enzyme		LVEF	= Left ventricular ejection fraction
AT	= Anaerobic threshold		NYHA	= New York Heart Association
BMI	= Body mass index		PETCO_2_	= End-tidal partial pressure of carbon dioxide
BTPS	= Body temperature pressure standard		PETO_2_	= End-tidal partial pressure of oxygen
CET	= Cardiopulmonary exercise test		RER	= Respiratory exchange ratio
CHF	= Chronic heart failure		RR	= Respiratory rate
CMDC	= Carbon monoxide diffusion capacity		SpO_2_	= Blood oxygen saturation
EOV	= Exercise oscillatory ventilation		SVC	= Slow vital capacity
FEV_1_	= Forced expiratory volume in 1 second		VCO_2_	= Carbon dioxide production
FV	= Flow-volume		VE	= Ventilation production
FVC	= Forced vital capacity		VO_2_	= Oxygen consumption
HR	= Heart rate			

## INTRODUCTION

Cardiovascular ischemic events are the leading cause of chronic heart failure (CHF), which is a syndrome that is generally characterized by the classic left ventricular systolic impairment with consequent muscular peripheral dysfunction^[1]^ caused by not only the low cardiac output, but also by medications, oxidative stress, and chronic hypoxemia, among others^[2]^. An important outcome of this peripheral muscular dysfunction is the reduced functional capacity, negatively affecting the patients' autonomy and consequently their quality of life^[2]^.

Many parameters are known as independent markers of severity and predictors of morbidity and mortality in this group of patients. The maximal inspiratory pressure has been shown as an independent variable to quantify the survival rate of these patients^[3]^ because it may reflect the inspiratory muscle weakness, usually witnessed in them. Furthermore, the handgrip strength has also been reported as an isolated parameter of CHF severity^[4]^. In this context, we may highlight the significance of the cardiopulmonary exercise test (CET). It is a useful tool that induce physiological responses in exercise conditions that might not appear at rest conditions.

From the parameters obtained in the CET, many of them have been described as negatively influenced by CHF progression. It is quite well known that patients with CHF present low functional status and exercise capacity, with reduced peak oxygen consumption (VO_2_)^[5,6]^. Another powerful CET variable that may reflect the severity of these patients and, more specifically, the pulmonary congestion is the ventilation production (VE)/ carbon dioxide production (VCO_2_) slope, which shows the ventilatory inefficiency, mainly in those who have values > 34, strongly characterizing pulmonary congestion^[7,8]^. Also, the presence of oscillatory ventilation in rest or during exercise is being considered as an important variable with prognostic value of CET^[9,10]^. Besides this importance, there is still no standardization for obtaining and interpreting exercise oscillatory ventilation (EOV)^[11]^.

Therefore, the aim of the present study is to characterize the presence of EOV and to relate it with other clinical variables in patients with CHF.

## METHODS

### Study Design

This is an observational and transversal study with convenience sample.

### Patients

Forty-six men with CHF were recruited by clinical assessment. Inclusion criteria were previous history of stable symptomatic CHF due to left ventricular systolic dysfunction, documented for at least six months (left ventricular ejection fraction [LVEF]: < 45%), New York Heart Association (NYHA) class between II-III, and clinical stability for at least three months. Patients were excluded from study if they had clinical and/or functional evidence of obstructive pulmonary disease (forced expiratory volume in 1 second [FEV_1_]/forced vital capacity [FVC] < 70% in pulmonary test)^[12]^, exercise-induced asthma, unstable angina or significant cardiac arrhythmias, and myocardial infarction within the previous six months; also, none of the subjects were tobacco users, alcohol dependents, or users of addicting drugs. No patient had been submitted to cardiovascular rehabilitation. All subjects presented the same clinical management, optimized medications, and were clinically stable. The eligible participants signed a written informed consent and the study protocol was approved by the Ethics Committee of Institution (protocol 238/06 and protocol 970.098).

### Experimental Procedure

The research was performed in an air-conditioned laboratory, with temperature between 22ºC and 24ºC, and relative humidity between 50 and 60%, always in the same period of the day (between 8 am and 12 pm). In the day before the test, patients were warned to avoid the intake of stimulating drinks, not to perform physical activity, and to have light meals and at least 8 hours of sleep. First, the volunteers were familiarized with the experimental set and involved researchers. Before the test begun, the patients were examined to verify if the recommendations were followed. Then, the systolic and diastolic arterial blood pressure and the peripheral oxygen saturation were measured, and it was performed auscultation.

### Pulmonary Function

Pulmonary function tests, measuring slow vital capacity (SVC), FVC, FEV_1_, and FEV_1_/FVC ratio, were carried out using the CardiO_2_ System (Medical Graphics Corporation, St. Paul, MO, USA). For comparative purposes, reference values from Knudson et al.^[13]^, expressed in body temperature pressure standard (BTPS) conditions, were used. Carbon monoxide diffusion capacity (CMDC) was assessed by simple respiration model and static volumes were assessed by whole-body plethysmography. Technical procedures and the acceptability and reproducibility criteria were defined according to norms recommended by the American Thoracic Society^[14]^.

### Ventilatory and Metabolic Variables During CET

Ventilatory and metabolic variables were obtained by a computer connected to an ergospirometric measurement system (CardiO_2_ System), using the Breeze Suite 6 software package. Tidal volume was obtained by a Pitot pneumotachometer connected to the CardiO_2_ System and attached to a facial mask - which was selected considering the volunteer's face size and providing an adequate fit in order to avoid air leakage. The device presents in real time applied power values (W) and pedaling speed (rpm), as well as VO_2_, VCO_2_, minute ventilation (VE), heart rate (HR), and blood oxygen saturation (SpO_2_). Ventilatory equivalent values (VE/VO_2_ and VE/VCO_2_), respiratory exchange ratio (RER), end-tidal partial pressure of oxygen (P_ET_O_2_) and carbon dioxide (P_ET_CO_2_), flow-volume (FV), and respiratory rate (RR) were also calculated and registered. The power applied to the cycle ergometer during exercise protocols was controlled by the system through an interface with the bicycle.

### Data Analysis

The following parameters were analyzed in CET variables:

#### First Ventilatory Threshold (At) Obtained

Visual analysis of VO_2_ and VCO_2_ correlation curves, VE/VO_2_, and P_ET_O_2_ were graphically represented in moving mean values each eight respiratory cycles. Subsequently, three independent observers determined the anaerobic threshold (AT) under the following situations: 1) V-slope: breaking point from linearity in VO_2_ and VCO_2_ correlation curves; 2) VE/VO_2_: nadir point of this ratio, ensuring that a systematic increase occurs from it; and 3) P_ET_O_2_: nadir point of this variable, from which a systematic increase begins. The CET data were set from the beginning of the ventilatory and metabolic variables responses to power output increments till the end of the exercise. Analysis of each observer was performed in an independent manner, on a 15 inches monitor (SyncMaster 550V, Samsung) connected to the MedGraphics software.

#### Exercise Oscillatory Ventilation (EOV)

The presence of periodic breathing was obtained by the analysis of ventilation data, and it was confirmed if there were three consecutive cycles with minimal average amplitude of 5 l in these data (peak value minus the average of two in-between consecutive nadirs), as suggested by Leite et al.^[15]^.

#### VE/VCO_2_ slope

VE and VCO_2_ data were analyzed from the beginning of the exercise till peak. Data were input into spreadsheet software (Microsoft Excel) to calculate VE/VCO_2_ slope via least squares linear regression (y = mx + b, m = slope).

VE/VCO_2_ > 34 and peak VO_2_ < 14 ml/kg/min were used to assess patients' severity.

### Statistical Analyses

Statistical analyses were performed using the SigmaPlot version 11.0.0.007 (for Windows(r)) with level of significance set at 0.05. Data were submitted to a normality test (Shapiro-Wilk). As a normal distribution was observed, parametric statistical tests were used. For intergroup comparisons, the t-Student pared test was applied. Demographics, anthropometrics, and clinical data were presented as means with standard deviation.

## RESULTS

**Fig. 1:**
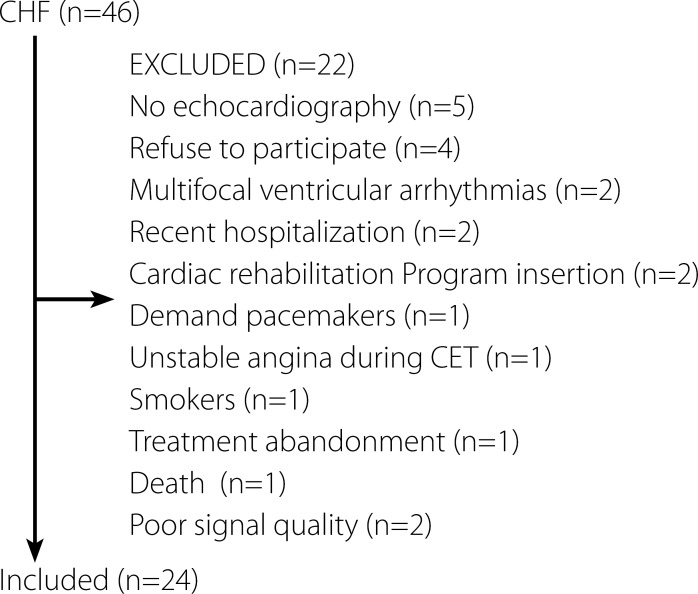
Flowchart of the present study. CHF = chronic heart failure; CET = cardiopulmonary exercise test.

Forty-six male patients were recruited; 22 patients were excluded and 24 were included in the present study (Figure 1).

Table 1 shows age and anthropometric and clinical characteristics of these patients, as well as their functional status and the CET variables with their prognostic thresholds. Body mass index (BMI) average showed that most of the patients were overweight and they were in NYHA functional class II and III. Among the 24 included patients, 16 presented EOV.

**Table 1:** Anthropometrics, clinical and cardiopulmonary exercise test (CET) data of the patients included in the present study.

Variables	(n=24)
Age (years)	53.1±13.6
Height (cm)	169±7
Mass (kg)	76.27±12.83
BMI (kg/m^2^)	26.5±3.8
Functional status	
NYHA II/III	9/15
CET	
Peak VO_2_ (ml/min)	1135.0±325.8
Peak VO_2_ (ml/kg/min)	15.1±4.1
AT VO_2_ (l/min)	655.8±189.1
AT VO_2_ (ml/kg/min)	8.7±2.5
Peak P_ET_CO_2_	29.7±7.8
VE/VCO_2_	40.1±15.7
EOV Yes/No	16/8
Prognostic values	
VE/VCO_2_ > 34 (n/%)	14/46
PeakVO_2_ < 14 ml/kg/min (n/%)	10/25
Medications	
Diuretics (n)	14
Digitalics (n)	9
Beta-blockers (n)	24
ACE-inhibitors (n)	15

Mean ± standard deviation. ACE=angiotensin-converting enzyme; AT=anaerobic threshold; BMI=body mass index; EOV=exercise oscillatory ventilation; NYHA=New York Heart Association; P_ET_CO_2_=end-tidal partial pressure of carbon dioxide; VE/VCO_2_=ventilation/carbon dioxide production; VO_2_=oxygen consumption

**Fig. 2:**
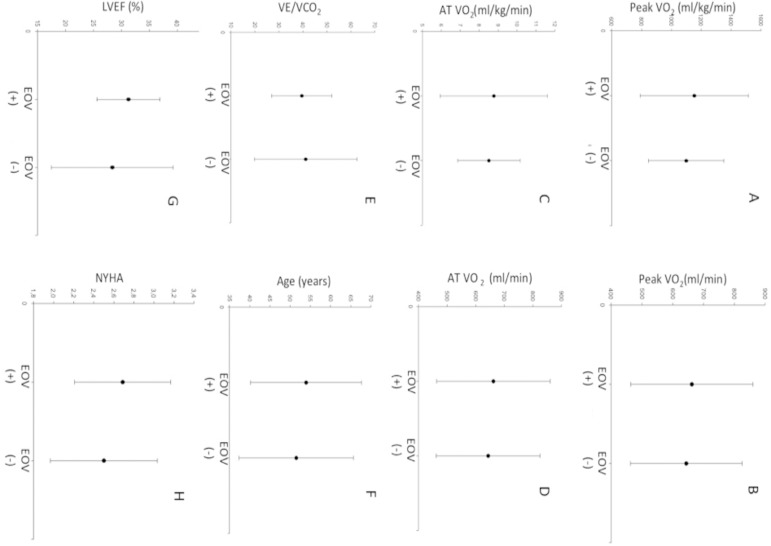
Analysis of age, cardiopulmonary exercise test and clinical variables of exercise oscillatory ventilation (EOV+) population and non-EOV (EOV-) population. (A) Peak oxygen consumption (ml/kg/min). (B) Peak oxygen consumption (ml/min). (C) Oxygen consumption (ml/kg/min) at anaerobic threshold (AT). (D) Oxygen consumption at AT (ml/min). (E) VE/VCO2 slope. (F) Age. (G) Left ventricular ejection fraction (LVEF) (%). (H) New York Heart Association (NYHA) functional class.

Figure 2 shows the data obtained from patients with EOV (EOV+) and patients who did not present EOV (EOV-) on incremental CET with other parameters obtained from the CET, as well as their clinical variables and age. There was no difference between EOV+ and EOV- groups.

Also, there was no difference on age, clinical variables and parameters of the CET data obtained from patients with EOV *and* VE/VCO_2_ slope > 34 when compared to patients with EOV *or* VE/VCO_2_ slope > 34, as shown on Table 2. Such analysis was performed to verify if the patients who presented these two concomitant responses had a more severe status than those who didn't.

**Table 2:** Analysis of cardiopulmonary exercise test (CET) parameters of patients with exercise oscillatory ventilation (EOV+) and VE/VCO_2_ >34 or just one of these variables.

	EOV (+) and VE/VCO_2_ >34	EOV (+) or VE/VCO_2_ > 34	*P* value
LVEF (%)	31.5±4.5	30±6.5	0.51
Peak VO_2_ (ml/kg/min)	13.8±3.7	16.1±5.1	0.25
Peak VO_2_ (ml/min)	1075.7±373.5	1199.0±349.9	0.47
AT VO_2_ (ml/kg/min)	8.3±2.3	9.4±3.1	0.39
AT VO_2_ (ml/min)	648.1±205.7	704.1±222.9	0.57
Age (years)	55.2±13.3	52.1±14.8	0.64
NYHA	2.7±0.5	2.6±0.5	0.68
Peak P_ET_O_2_ (mmHg)	109.1±4.3	104.9±7.4	0.13
Peak P_ET_CO_2_ (mmHg)	26.±3.7	31.±10.3	0.34
Peak workload (W)	84.9±27.6	91.3±31.0	0.64
HR (bpm)	123.5±27.0	122.5±19.8	0.92

Mean ± standard deviation. *P* value < 0.05

AT=anaerobic threshold; HR=heart rate; LVEF=left ventricle ejection fraction; NYHA=New York Heart Association; PetCO_2_=end-tidal partial pressure of carbon dioxide; P_ET_O_2_=end-tidal partial pressure of oxygen; VE/VCO_2_=ventilation/carbon dioxide production; VO_2_=oxygen consumption

## DISCUSSION

The present study's main findings are: (i) no difference between CET variables in patients with EOV and non-EOV; (ii) the absence of difference on functional variables between CHF patients with EOV *and* VE/VCO_2_ > 34 and those with EOV *or* VE/VCO_2_ > 34; iii) the presence of EOV and peak VO_2_ < 14 ml/kg/min in 25% of the patients, as well as EOV and VE/VCO_2_ > 34 in 46% of them.

Anthropometric data of CHF patients showed that they were overweight and that 15 of the 24 evaluated patients were classified as NYHA functional class III. Furthermore, they had a poor exercise performance on CET, which can be seen by the value of peak VO_2_ (15.1 ml/kg/min), presenting low peak workload values. The literature shows that CHF patients exhibit a low peak VO_2_^[16]^ as a marker of exercise intolerance caused by many factors of this disease as the low cardiac output, pulmonary congestion, and alterations of metabolism on peripheral and ventilatory muscle fibers that lead to a muscular dysfunction with impact on exercise tolerance. Peak VO_2_ is also a prognostic variable of CET^[5,6]^.

Other CET variables showed similarities between our study and the literature, specifically when it comes to the coexisting presence of EOV and other bad prognostic variables, such as VE/VCO_2_ > 34 and peak VO_2_ < 14 ml/kg/min^[17]^. One study has showed that the presence of the combination EOV and VE/VCO_2_ > 34 is particularly more alarming because of the risk for adverse cardiac events^[18]^. Otherwise, our results do not agree with the literature when it comes to the worst response of CET variables on EOV population, described as lower peak VO_2_, higher VE/VCO_2_ slope, and lower rest and peak P_ET_CO_2_, when compared to non-EOV population^[17]^. Hypotheses for these findings are the heterogeneity of exercise protocols in the literature and the absence of a gold standard to verify the presence of EOV in patients with profile and clinical status similar to our subjects. In a meta-analysis about the assessment of EOV, Cornelis et al.^[11]^ have suggested the use of Corrà et al.^[19]^ criteria, although none of the criteria available appears to be superior. This criterion should be applied to a constant load protocol since the VE data may not vary more than 15% compared to the mean of rest VE data, which is a physiological response expected on the incremental exercise protocol. Also, the presence of EOV may be longer than 60% of the exercise time. For the results presented, we used Leite et al.^[15]^ criteria because these are not so subjective since the presence of EOV is not calculated through time, but as a continuous variation of the VE data with an waxing and waning pattern, and it is not influenced by the time of its appearance, but by its amplitude. Finally, we believe that Leite et al.^[15]^ criteria could be more appropriate to assess EOV during incremental exercise test.

Even though the trigger mechanisms of EOV are not totally understood, the main hypotheses are circulatory delay, increased chemosensivity, increased ergoreflex signaling, and/or pulmonary congestion^[17]^. The reduced cardiac output leads to a delayed lung-chemoreceptor circulation (peripheral/central); this and the inefficient control of VE caused by increased chemosensitivity lead to an exaggerated response of the ventilation^[20]^. From the hemodynamic view, there is an uncoupling on the right ventricle to lung circulation, and a pulmonary edema due to a high ventricle filling pressure even when these patients are clinically stable and on optimized drug therapy^[20]^.

Studies that evaluated the prognostic power of EOV obtained by the analysis of ventilatory pattern of CHF patients when submitted to a CET suggest that this variable seems to be the most important in the CET, even with better prognostic values than VE/VCO_2_ slope^[21]^. Additionally, the presence of EOV combined with higher values of VE/VCO_2_, mainly > 34, is even more alarming and powerful to predict adverse cardiac events on CHF population^[18]^, characterizing that these two ventilatory variables reflect the worst control on ventilation and ventilatory inefficiency and may be translated into a better prognostic definition^[20]^. In the present study, the comparison between patients with EOV and VE/VCO_2_ > 34 and patients with only one of these showed no statistical difference on CET ventilatory variables, age, nor their clinical status, such as LVEF. It is already known that the presence of EOV is not altered by LVEF, since Guazzi et al.^[22]^ showed no difference in incidence of EOV in CHF patients with normal or reduced LVEF. It suggests the power of EOV as an independent CET marker of worst prognostic because it represents the poor hemodynamic and ventilatory adjust to physical exercise and did not correlate with other ventilatory and metabolic CET variables with prognostic values.

Some studies focused on treatment of EOV and showed that the pathological pattern of ventilation in EOV population can be modulated and even disappear. Three studies evaluating pharmacological therapy with inodilator (malrinone)^[23]^ and selective pulmonary vasodilator (sildenafil)^[24]^ have shown some attenuation on EOV. In another study based on aerobic training, for three months, 71% of the patients with stable congestive heart failure showed a good EOV response^[25]^. These studies evaluated stable patients on optimized drug therapy, which suggests that maybe EOV does not respond to standard treatment for CHF, requiring other approaches than pharmacological interventions, such as physical exercise.

Based on the present study's findings, it is important to encourage further studies about EOV in CHF and other patients for a better comprehension of the role of EOV, as well as to establish a gold standard pattern to verify the presence of this variable in different diseases and levels of severity. Finally, this knowledge improves therapeutic strategies.

### Limitations

The absence of gold standard in obtaining EOV must be considered, also some tool to evaluate peripheral muscular strength would give information that could help the interpretation of the findings. Results may not be extrapolated to more severe patients. Finally, this study was made with a convenience sample and more subjects should be recruited to consolidate our findings.

## CONCLUSION

The present study showed that there was an incidence of patients with EOV and lower peak VO_2_ and higher VE/VCO_2_ slope values, but there was no difference on other prognostic variables. In addition, no influence of the EOV presence on other parameters of CET in this population was observed, suggesting that this CET variable may be an independent marker of severity in CHF patients.

**Table:** 

Authors' roles & responsibilities	
HVR	Substantial contributions to the conception or design of the work; or the acquisition, analysis, or interpretation of data for the work; final approval of the version to be published
PAS	Final approval of the version to be published
CLC	Final approval of the version to be published
SG	Final approval of the version to be published
JAN	Final approval of the version to be published
ABS	Final approval of the version to be published
MSR	Substantial contributions to the conception or design of the work; or the acquisition, analysis, or interpretation of data for the work; final approval of the version to be published

## References

[1] Dempsey JA, Romer L, Rodman J, Miller J, Smith C (2006). Consequences of exercise-induced respiratory muscle
work. Respir Physiol Neurobiol.

[2] Gosker HR, Wouters EF, van der Vusse GJ, Schols AM (2000). Skeletal muscle dysfunction in chronic obstructive pulmonary
disease and chronic heart failure: underlying mechanisms and therapy
perspectives. Am J Clin Nutr.

[3] Cahalin LP, Arena R, Guazzi M, Myers J, Cipriano G, Chiappa G (2013). Inspiratory muscle training in heart disease and heart failure: a
review of the literature with a focus on method of training and
outcomes. Expert Rev Cardiovasc Ther.

[4] Izawa KP, Watanabe S, Osada N, Kasahara Y, Yokoyama H, Hiraki K (2009). Handgrip strength as a predictor of prognosis in Japanese
patients with congestive heart failure. Eur J Cardiovasc Prev Rehabil.

[5] Arena R, Myers J, Guazzi M (2008). The clinical and research applications of aerobic capacity and
ventilatory efficiency in heart failure: an evidence-based
review. Heart Fail Rev.

[6] Gibbons RJ, Balady GJ, Beasley JW, Bricker JT, Duvernoy WY, Froelicher VF (1997). ACC/AHA Guidelines for Exercise Testing. A report of the American
College of Cardiology/American Heart Association Task Force on Practice
Guidelines (Committee on Exercise Testing). J Am Coll Cardiol.

[7] Poggio R, Arazi HC, Giorgi M, Miriuka SG (2010). Prediction of severe cardiovascular events by VE/VCO2 slope
versus peak VO2 in systolic heart failure: a meta-analysis of the published
literature. Am Heart J.

[8] Arena R, Myers J, Aslam SS, Varughese EB, Peberdy MA (2004). Peak VO2 and VE/VCO2 slope in patients with heart failure: a
prognostic comparison. Am Heart J.

[9] Cahalin LP, Chase P, Arena R, Myers J, Bensimhon D, Peberdy MA (2013). A meta-analysis of the prognostic significance of cardiopulmonary
exercise testing in patients with heart failure. Heart Fail Rev.

[10] Arena R, Myers J, Abella J, Peberdy MA, Pinkstaff S, Bensimhon D (2008). Prognostic value of timing and duration characteristics of
exercise oscillatory ventilation in patients with heart
failure. J Heart Lung Transplant.

[11] Cornelis J, Beckers P, Vanroy C, Volckaerts T, Vrints C, Vissers D (2015). An overview of the applied definitions and diagnostic methods to
assess exercise oscillatory ventilation: a systematic review. Int J Cardiol.

[12] Pauwels RA, Buist AS, Calverley PM, Jenkins CR, Hurd SS, GOLD Scientific Committee (2001). Global strategy for the diagnosis, management, and prevention of
chronic obstructive pulmonary disease. NHLBI/WHO Global Initiative for
Chronic Obstructive Lung Disease (GOLD) Workshop summary. Am J Respir Crit Care Med.

[13] Knudson RJ, Lebowitz MD, Holberg CJ, Burrows B (1983). Changes in the normal maximal expiratory flow-volume curve with
growth and aging. Am Rev Respir Dis.

[14] American Thoracic Society (1995). Standardization of Spirometry, 1994 Update. Am J Respir Crit Care Med.

[15] Leite JJ, Mansur AJ, Freitas HF, Chizola PR, Bocchi EA, Terra-Filho M (2003). Periodic breathing during incremental exercise predicts mortality
in patients with chronic heart failure evaluated for cardiac
transplantation. J Am Coll Cardiol.

[16] Arena R, Guazzi M, Cahalin LP, Myers J (2014). Revisiting cardiopulmonary exercise testing applications in heart
failure: aligning evidence with clinical practice. Exerc Sport Sci Rev.

[17] Cornelis J, Taeymans J, Hens W, Beckers P, Vrints C, Vissers D (2015). Prognostic respiratory parameters in heart failure patients with
and without exercise oscillatory ventilation: a systematic review and
descriptive meta-analysis. Int J Cardiol.

[18] Guazzi M, Arena R, Ascione A, Piepoli M, Guazzi MD, Gruppo di Studio Fisiologia dell'Esercizio, Cardiologia dello Sport
e Riabilitazione Cardiovascolare of the Italian Society of
Cardiology (2007). Exercise oscillatory breathing and increased ventilation to
carbon dioxide production slope in heart failure: an unfavorable combination
with high prognostic value. Am Heart J.

[19] Corrà U, Giordano A, Bosimini E, Mezzani A, Piepoli M, Coats AJ (2002). Oscillatory ventilation during exercise in patients with chronic
heart failure: clinical correlates and prognostic
implications. Chest.

[20] Guazzi M (2014). Abnormalities in cardiopulmonary exercise testing ventilatory
parameters in heart failure: pathophysiology and clinical
usefulness. Curr Heart Fail Rep.

[21] Sun XG, Hansen JE, Beshai JF, Wasserman K (2010). Oscillatory breathing and exercise gas exchange abnormalities
prognosticate early mortality and morbidity in heart failure. J Am Coll Cardiol.

[22] Guazzi M (2012). Treating exercise oscillatory ventilation in heart failure: the
detail that may matter. Eur Respir J.

[23] Ribeiro JP, Knutzen A, Rocco MB, Hartley LH, Colucci WS (1987). Periodic breathing during exercise in severe heart failure.
Reversal with milrinone or cardiac transplantation. Chest.

[24] Murphy RM, Shah RV, Malhotra R, Pappagianopoulos PP, Hough SS, Systrom DM (2011). Exercise oscillatory ventilation in systolic heart failure: an
indicator of impaired hemodynamic response to exercise. Circulation.

[25] Zurek M, Corrà U, Piepoli MF, Binder RK, Saner H, Schmid JP (2012). Exercise training reverses exertional oscillatory ventilation in
heart failure patients. Eur Respir J.

